# Genetic Basis of Variations in Nitrogen Source Utilization in Four Wine Commercial Yeast Strains

**DOI:** 10.1371/journal.pone.0067166

**Published:** 2013-06-24

**Authors:** Alicia Gutiérrez, Gemma Beltran, Jonas Warringer, Jose M. Guillamón

**Affiliations:** 1 Departamento de Biotecnología de los alimentos, Instituto de Agroquímica y Tecnología de los Alimentos (CSIC), Paterna (Valencia), Spain; 2 Departament de Bioquímica i Biotecnologia, Facultat d’Enologia, Universitat Rovira i Virgili. Tarragona, Spain; 3 Department of Chemistry and Molecular Biology, University of Gothenburg, Gothenburg, Sweden; University of Strasbourg, France

## Abstract

The capacity of wine yeast to utilize the nitrogen available in grape must directly correlates with the fermentation and growth rates of all wine yeast fermentation stages and is, thus, of critical importance for wine production. Here we precisely quantified the ability of low complexity nitrogen compounds to support fast, efficient and rapidly initiated growth of four commercially important wine strains. Nitrogen substrate abundance in grape must failed to correlate with the rate or the efficiency of nitrogen source utilization, but well predicted lag phase length. Thus, human domestication of yeast for grape must growth has had, at the most, a marginal impact on wine yeast growth rates and efficiencies, but may have left a surprising imprint on the time required to adjust metabolism from non growth to growth. Wine yeast nitrogen source utilization deviated from that of the lab strain experimentation, but also varied between wine strains. Each wine yeast lineage harbored nitrogen source utilization defects that were private to that strain. By a massive hemizygote analysis, we traced the genetic basis of the most glaring of these defects, near inability of the PDM wine strain to utilize methionine, as consequence of mutations in its *ARO8*, *ADE5,7* and *VBA3* alleles. We also identified candidate causative mutations in these genes. The methionine defect of PDM is potentially very interesting as the strain can, in some circumstances, overproduce foul tasting H_2_S, a trait which likely stems from insufficient methionine catabolization. The poor adaptation of wine yeast to the grape must nitrogen environment, and the presence of defects in each lineage, open up wine strain optimization through biotechnological endeavors.

## Introduction

Inoculation of selected yeast into wine must, rather than relying on spontaneous fermentation, is an established oenological practice that allows better control of organoleptic wine characteristics and guarantees the homogeneity of successive fermentations. Nowadays, most commercial wine production is based on such commercial starter wine yeasts, which were originally selected mainly from natural varieties of the Wine/European genetic clade [1], given their superior fermentation properties. However, the overall suitability of wine yeasts to grape wine production, which imposes demands for a large number of genetically complex traits, has not been stringently evaluated. The vast variability among natural yeasts [2], in combination with widespread antagonistic pleiotropy, suggests that any one strain selected from a natural stock is unlikely to possess an ideal combination of oenological characteristics. It is also unclear as to what extent wine strains have adapted to wine production conditions; for example, many wine strains are poor at utilizing proline, the predominant nitrogen source in grape wine, despite undergoing nitrogen limitation during wine fermentation [3]. Thus, it is easy to envision a substantial potential for optimization of existing wine yeasts.

Nitrogen source utilization has a substantial impact on alcoholic fermentation, influencing both the fermentative process and wine quality [4], [5]. Nitrogen deficiency can produce sluggish or stuck fermentations, and both nitrogen deficiency and incomplete nitrogen utilization can confer poor organoleptical properties. Conversely, excessively high nitrogen levels may have negative effects, such as microbial contamination, production of off-flavors [6] or ethyl carbamate formation, which is a suspected carcinogen [7]. Thus, there is particular interest in optimizing wine yeast nitrogen utilization in a way that ensures that all the nitrogen compounds present in the grape must are utilized completely and efficiently. Common lab strains of *Saccharomyces cerevisiae* can catabolize a variety of low complexity organic nitrogen sources, such as most amino acids, the animal secretion products urea and allantoin, the arginine derivative citrulline, some nitrogen bases and one inorganic nitrogen source, ammonium [8]. These compounds enter cells via permeases and are rapidly used as building blocks in biosynthesis or are catabolized to yield the internal nitrogen currencies ammonium or glutamate [9]. In complex mixtures of nitrogen compounds, wine yeast prefers utilizing certain sources before others, and this pattern of nitrogen compound uptake depends on both nitrogen and sugar composition [10], [11]. In the presence of a single nitrogen source, neither nitrogen source preference nor the achieved growth rate has been exhaustively mapped across wine strains. Nevertheless, based on lab strain experiments, it is commonly assumed that preferred nitrogen sources allow higher growth rates. In lab strains, nitrogen source preference is mediated by the nitrogen catabolite repression (NCR) system by stimulating the expression of permeases for the preferred nitrogen source and the degradation of permeases of non preferred sources [12]. Ammonium, glutamine and asparagine are preferred nitrogen sources whereas arginine, alanine, aspartate and glutamate are less preferred, and urea and proline non preferred [12], [13]. Branched-chain and aromatic amino acids do not support high growth rates, but typically accumulate early in fermentation [10], [14], thus breaking the assumed correlation between the nitrogen source growth rate and preference.

A primary challenge for the human-induced improvement of nitrogen-associated properties of wine yeast is the dissection of the genetic architectures underlying variations in the capacity to utilize nitrogen sources between commercially established wine strains. The yeast universal reference strain S288c, and its relative Σ1278b, on which much molecular understanding of the nitrogen metabolism is based, are phenotypically much diverged from wine yeasts [2]. Thus, only a limited extrapolation of knowledge from lab strain experimentation is possible, and the associations between variation in nitrogen utilization traits and genetic variation have to be established in wine strains without prior assumptions. Yeast has recently emerged as a prime model organism for quantitative genetics in general and for Quantitative Trait Loci (QTL) mapping in particular [15]. Variations in high temperature growth [16], [17], sporulation efficiency [18], drug response [19], [20], telomere homeostasis [21], cell morphology [22], ethanol tolerance [23] and acetic acid production [24] have all been mapped to individual genes. More recently, Salinas *et al.* [25] and Ambroset *et al*. [26] identified QTLs of oenological phenotypes. However, the genetic basis of trait variations in commercially relevant strains have been dissected only in a very small number of cases [24], [26], [27].

Here, we precisely and exhaustively quantified variations in the ability of four widely used commercial wine strains in Spanish wineries to utilize the complete palette of low complexity nitrogen sources that is normally accessible to yeast. We report extensive growth differences between nitrogen sources and different wine strains. Some of these differences in growth are present in all the lineages, while other variations are nitrogen defects that are private to each strain. We traced the genetic origin of the incapacity of the PDM wine strain to utilize methionine to defects in its *ARO8*, *ADE5,7* and *VBA3* alleles, which is of particular interest as these defects may contribute to an excessive production of foul tasting H_2_S in this strain. Finally, we suggest specific nucleotides that can be targeted in efforts to alleviate this deficiency.

## Materials and Methods

### Yeast Strains and Media

The yeast strains used in this study are the following: PDM, ARM, RVA and TTA; all of which were provided by Agrovin Company (Ciudad Real, Spain). The oenological features of these strains can be obtained from the company web page (http://www.agrovin.com). A taxonomic description of these strains was carried out by the RFLPs of the ITS/5.8S region [28]. Strains PDM (Pasteur Prise de Mousse), RVA and TTA belong to species *Saccharomyces cerevisiae*, while we identified strain ARM as a hybrid between *S. cerevisiae* and *S. kudriavzevii*, following the procedure proposed by Gonzalez *et al*. [29]. This latter strain is commercialized by Maurivin as EP2 and its hybrid nature has recently been confirmed by Dunn *et al*. [30]. These wine strains were grown at 30°C on plates of YPD medium (2% glucose, 1% yeast extract, 1% peptone and 2% agar).

The synthetic wine must (SWM) was prepared according to Riou *et al*. [31], but with 200 g/L of reducing sugars (100 g/L glucose +100 g/L fructose) and without anaerobic factors [32]. Only the nitrogen content changed. Each medium was prepared with a single nitrogen source, except for the control condition (SWMc), which was composed of a mixture of ammonium and amino acids (40% of ammonium +60% of amino acids), as described in Beltran *et al*. [32]. The nitrogen sources used were: adenine, allantoin, ammonium, cytosine, GABA, L-alanine, L-arginine, L-asparagine, L-aspartate, L-citrulline, L-glutamate, L-glutamine, L-isoleucine, L-leucine, L-methionine, L-ornithine, L-phenylalanine, L-proline, L-serine, L-threonine, L-tryptophan, L-valine, L-urea. The tested concentrations were 30 mg N/L as a highly nitrogen deficient condition and 140 mg N/L as a control condition, which resembles the more realistic nitrogen concentrations in wine must.

### Measure of Growth Variables

Two consecutive pre-cultures of 72 hours were performed by incubating cells at 30°C in 350 µL of SWM medium with 30 mg/L of ammonium as the sole nitrogen source in 100-well micro-cultivation plates. This low concentration is required for all the strains to deplete their nitrogen reserves; thus enabling the test of their utilization of different nitrogen sources, starting from the same initial cellular state. Asexual reproduction was monitored at 600 nm in a Bioscreen analyser C (Thermo Labsystems Oy, Finland). Pre-cultures were inoculated at an initial OD of approximately 0.1 (inoculum level of 10^6^ CFU/mL) in the SWM with different nitrogen concentrations and sources. Incubation was maintained at 30°C (10 min preheating time). Microcultivation plates were subjected to shaking at the highest shaking intensity with 60 s of shaking every other minute. OD measurements were taken every 20 min over a 72-hour period. This time allows yeast cells to reach the stationary phase in all but the worst nitrogen environments. All the conditions were run in duplicate at both nitrogen concentrations. In all, 384 growth curves (24 nitrogen sources×2 nitrogen concentrations×4 yeast strains×2 replicates) were obtained and analyzed. For each growth curve, the variables lag phase, doubling time and growth efficiency were extracted as described [33]. Briefly, the lag phase was estimated using the slope calculation from every eight consecutive data values along the curve (corresponding to a time span of 2.5 h). An intercept between every slope and a straight line corresponding to the initial OD was calculated. A mean of the two highest calculated intercepts was taken as the lag phase. Generation time was calculated by taking into account the slopes between every third consecutive measurements for the whole growth curve. Of the seven highest slopes, the highest two were discarded to provide a safety margin, and a mean was calculated for the remaining five. The generation time was obtained as ln 2 divided by the mean of the slopes. Growth efficiency was calculated based on the six last time points in the measurement. The difference between end OD and initial OD was taken as the stationary phase OD increment.

### Construction of Haploid Strains and Mating Type Determination

To carry out the construction of derivative haploid wine strains, the *HO* gene was deleted in the PDM diploid strain using the short flanking homology method reported by Güldener *et al*. [34]. This method replaces one copy of the open reading frame of *HO* gene with the *nat*MX4 cassette. The deletion cassette was obtained by PCR using the pAG25 plasmid that contains nourseothricin resistance. The primers used, HO-S1 and HO-C2 (Table S1), have 50 nucleotide extensions corresponding to the regions upstream of the target gene start codon (forward primer) and downstream of the stop codon (reverse primer). PDM strain was transformed by the lithium acetate procedure [35]. Transformants were selected by resistance to nourseothricin and correct integration of the deletion cassette was confirmed by diagnostic PCR using the primers upstream and downstream of the deleted region (Table S1).

Sporulation was induced by incubating cells on acetate medium (1% potassium acetate and 2% agar) for 5 days at 30°C. Following the preliminary digestion of ascus walls with 2 mg/ml glucuronidase (Sigma), spores were dissected using micromanipulation (Singer instruments, United Kingdom). In all cases, >50% of spores were viable. Finally, monosporic cultures were grown on YPD plates in the presence of nourseothricin. To test the mating type of each haploid strain selected, PCR against the *MAT* locus was performed using *MAT* ***a*** and *MAT*α primers [36] (Table S1). Both the 544 bp haploid *MAT* ***a*** and the 404 bp haploid *MAT*α bands were observed in the diploid strains. PCR was done under the following conditions: 94°C for 5 min, 30 cycles at 94°C for 1 min, 58°C for 2 min and 72°C for 2 min, and 72°C for 7 min. Haploid strains were grown under the same nitrogen conditions as their diploid parent strains.

### Hemizygosity Analysis

To identify the alleles contributing to variations in nitrogen source utilization, 228 hemizygote hybrids, each resulting from a cross between a derivative haploid PDM strain and a BY4741 derivative lacking one of the 228 nitrogen utilization genes, were constructed. The haploid of the PDM wine strain (*MAT* **α**; *ho*Δ) was crossed with the deletion mutants from the BY4741 deletion collection (*MAT* **a**; *his3*Δ*1*; *leu2*Δ*0*; *met15*Δ*0*; *ura3*Δ*0*). Table S2 lists all the deletion strains used. A heterozygote hybrid strain, which also maintained the BY gene, was also constructed and used as a control. Constructions were performed as follows: haploid strains were grown in 96-well plates with liquid YPD media for 24 h at 30°C. The wine and BY strain cultures were spotted onto the same positions on solid YPD medium in 96-well format dishes using a benchtop RoToR HDA robot (Singer Instruments, United Kingdom) with default settings. After 48 h at 30°C, colonies were re-pinned onto similar YPD 96-well format dishes supplemented with 0.2 mg/mL geneticin and 0.05 mg/mL nourseothricin resistance to select the diploid hybrids from successful matings. Strains were manually transferred to 100-well bioscreen microcultivation and plates, and were grown in SWM with selected nitrogen sources. Growth was quantified as indicated above and comparing each hemizygote to the heterozygote diploid control (n = 5).

### Construction of the Haploid Deletion Mutants in the PDM Wine Strain

*ARO8, BAT2, ADE5,7, VBA3* were independently deleted in the haploid derivative of the PDM wine strain using a short flanking homology [34]. The deletion cassette contained hygromycin B resistance, amplified from plasmid pAG32 with the primers shown in Table S1. Primers had 50-nucleotide extensions corresponding to the regions upstream and downstream of the target ORF and were transformed into the haploid wine strain following the lithium acetate procedure [35]. For each construct, three transformants resistant to hygromycin B were analyzed by diagnostic PCR and were used as independent repeats (n = 3). Wine deletion strains were crossed to the BY4741 strain on YPD, and diploids were selected on the medium containing 0.2 mg/mL geneticin and 0.3 mg/mL hygromycin B. Once again, these hemizygotes were grown in SWM with selected nitrogen sources, as described above, and were compared to each respective reciprocal hemizygote missing the BY allele. The growth of these hemizygotes was also performed in 50 mL tubes to check their phenotype under conditions more similar to real wine production.

### Sequence Analysis

Four genes (*ARO8*, *ADE5,7*, *BAT2* and *VBA3*) were sequenced in the wild-type strain PDM by Macrogen Inc. facilities (Seoul, South Korea) using an ABI3730 XL automatic DNA sequencer. The primers designed for PCR amplification are shown in Table S1.

### Clustering Methods and Statistical Analysis

A two-tailed Student’s t-test with equal variance assumption was used for the two-group comparisons. The cut-off level of significance was set to α ≤0.05. Hierarchical clustering was performed using MeV MultiExperiment Viewer, and Pearson correlation metrics and group clustering based on group averages (average linkage). Pearson correlation coefficients were employed for the correlation analysis. Significance of the correlations was calculated using a Student’s t-distribution, 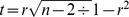, where *r* = Pearson correlation coefficient and *n* = number of nitrogen sources. Degrees of freedom = *n*−2.

## Results

### Different Wine Yeast Growth Measures Provide Complementary Views of Nitrogen Source Suitability

**Figure 1:**
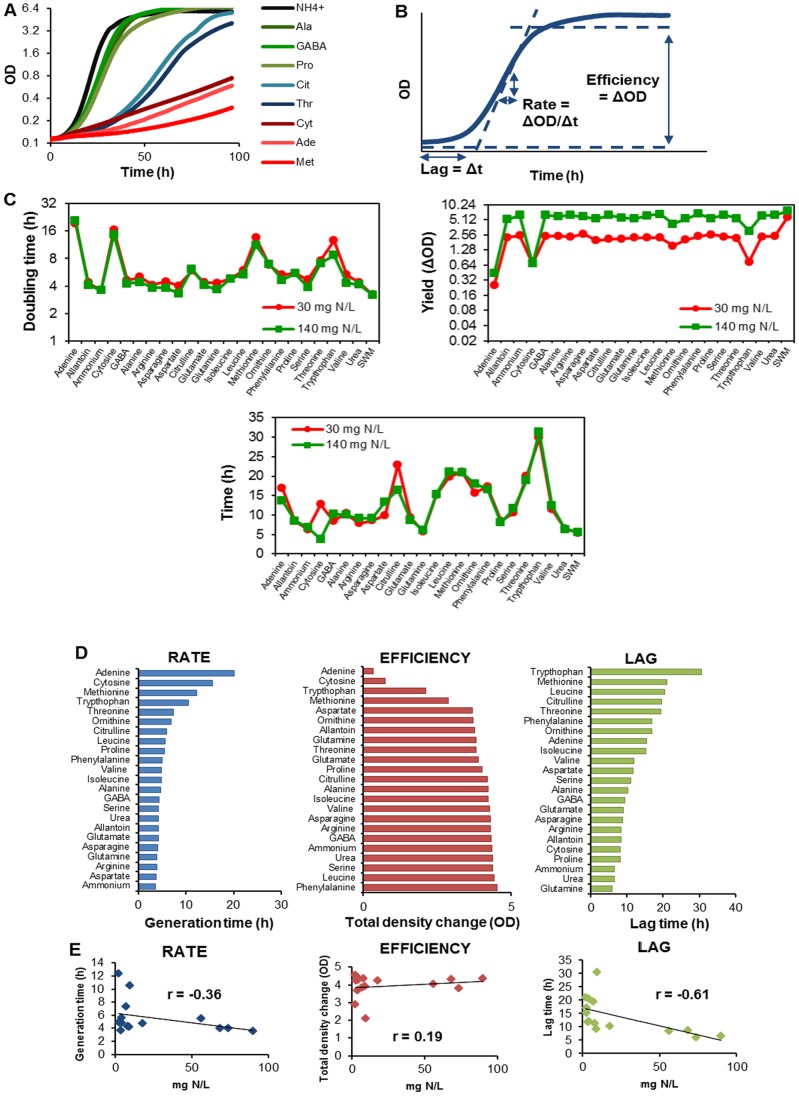
Vast variations between wine yeasts in nitrogen source utilization capacity. The capacity of four wine yeasts to utilize low-complexity nitrogen compounds as sole nitrogen sources was quantified using microcultivation and extraction of asexual fitness components from high density mitotic growth curves. A palette of 24 nitrogen sources was tested at low (30 mg N/mL) and intermediate (140 mg N/mL) concentrations. A) Sample mitotic growth curves of the PDM strain in a subset of nitrogen sources. B) The asexual fitness components lag phase (time to initiate asexual proliferation), rate (asexual generation time) and efficiency (total change in population density during asexual growth) were extracted from each high density growth curve. C) Effect of the concentration of nitrogen on the mean (n = 2) of the asexual fitness components rate, efficiency and lag of the four wine strains. D) The mean of each fitness component measure (n = 2 for each strain), over all four wine strains, was calculated. Nitrogen sources were then ranked separately according to mean performance for each fitness component. E) The mean in performance between the four wine strains was plotted against amount of nitrogen of each particular source present at the complete SWMc (control condition). Linear regression (black line) is displayed. The squared Pearson correlation coefficient (r) is provided in the figure.

To quantify variations in the nitrogen source utilization among wine strains, strains PDM, ARM, RVA and TTA were microcultivated at low and intermediate concentrations (30 and 140 mg N/L) of 23 individual nitrogen substrates. Together, these substrates covered the entire width of the low complexity nitrogen compounds utilizable as sole nitrogen sources by the yeast lab strain S288c. High density mitotic growth curves revealed vast variations in the capacity of the wine strains to utilize different nitrogen sources (Fig. 1A), which did not immediately agree with the established wisdom on nitrogen source preference. To obtain quantitative measures of mitotic performance and to allow a stringent evaluation, lag phase length (lag), the exponential growth rate (doubling time) and growth efficiency (total change in density) were extracted (Fig. 1B). Together, these measures encapsulate yeast mitotic fitness. Overall, the tested (low or intermediate) nitrogen concentrations were found irrelevant for the lag and rate of wine strain mitotic proliferation (Fig. 1C). In contrast, the efficiency of all the nitrogen sources, excluding the very poor adenine and cytosine, which were not exhausted within the experimental time frame, were very strongly affected by nitrogen availability (Fig. 1C). Thus, nitrogen availability was limiting for the biomass yield, but not for the rate or the lag of biomass production. This result is in agreement with recent findings [37]. The mean difference in efficiency between nitrogen concentration (excluding adenine and cytosine) ranged from 3.85 (PDM) to 3.57 (TTA), which is reasonably close to the theoretical expectation of (4.66-fold) by assuming strictly additive effects of nitrogen increase on yield within the concentration range considered.

To analyze the effect of the different nitrogen sources on the three mitotic fitness measures in wine strains, the average growth data of the four strains were used to establish a ranking of these nitrogen compounds in terms of their rate, efficiency (yield) and lag (Fig. 1D). The relative capacity of different nitrogen sources to support wine yeast proliferation diverged from the accepted view of nitrogen source suitability for lab strains. Furthermore, the different fitness measures only partially overlapped [38]. As expected, a ranking of the nitrogen sources based on the mean growth rate showed that: nucleotide bases were very poor nitrogen sources; aromatic and branched amino acids, together with ornithine and citrulline, were poor nitrogen sources; the nitrogen sources traditionally classified as preferred supported fast or very fast growth (average generation time ∼ 4 h) (Fig. 1D). Surprisingly however, methionine supported only very slow growth in wine strains, whereas urea and allantoin promoted fast reproduction. The two latter compounds have been traditionally classified as poor sources that do not exert an NCR effect [38]. In contrast, the efficiency measure, arguably the most relevant for wine production, revealed that several nitrogen sources traditionally regarded as poor, notably phenylalanine, leucine and citrulline, were very efficiently utilized, whereas nitrogen sources traditionally classified as good, such as aspartate, glutamine and glutamate, were less optimal in utilization efficiency terms. Finally, the ranking of lag phase lengths revealed that urea, proline, ammonium and glutamine were metabolized with a short delay, whereas tryptophan, leucine, methionine and citrulline required almost one full re-adjustment day before allowing proliferation to take off. Thus, the different growth measures provided complementary views of nitrogen source suitability, which deviated from what has been formerly been established using lab strains. This underscored the importance of weighing different aspects of mitotic growth when judging nitrogen source suitability as well as the limitations of extrapolations from lab to wine strains.

The capacity of a particular nitrogen source to support fast or efficient yeast growth showed no correlation whatsoever to the abundance of this nitrogen compound in grape must (Fig. 1E). For example, wine strains were excellent at utilizing urea and allantoine, which are absent in grape must, but proved to be slower and less efficient in utilizing very abundant nitrogen compounds, such as proline. This casts doubts on the assumption that yeast in general, and wine yeast in particular, are well-adapted to grape must. Surprisingly, lag phase length, a trait which has received little attention in wine production and in yeast research, showed a strong inverse correlation (Pearson, r = –0.69, p = 0.01) to nitrogen abundance. That is, lag phase length was much shorter when wine yeasts were adjusting their metabolism to the nitrogen compounds that are abundant in grape must. Taken together, human domestication of yeast for wine production appears to leave the rate and efficiency of nitrogen source utilization unaffected, but may substantially shorten the lag before this utilization takes off.

### Wine Strains Differ in their Capacity to Utilize Different Nitrogen Sources

**Figure 2:**
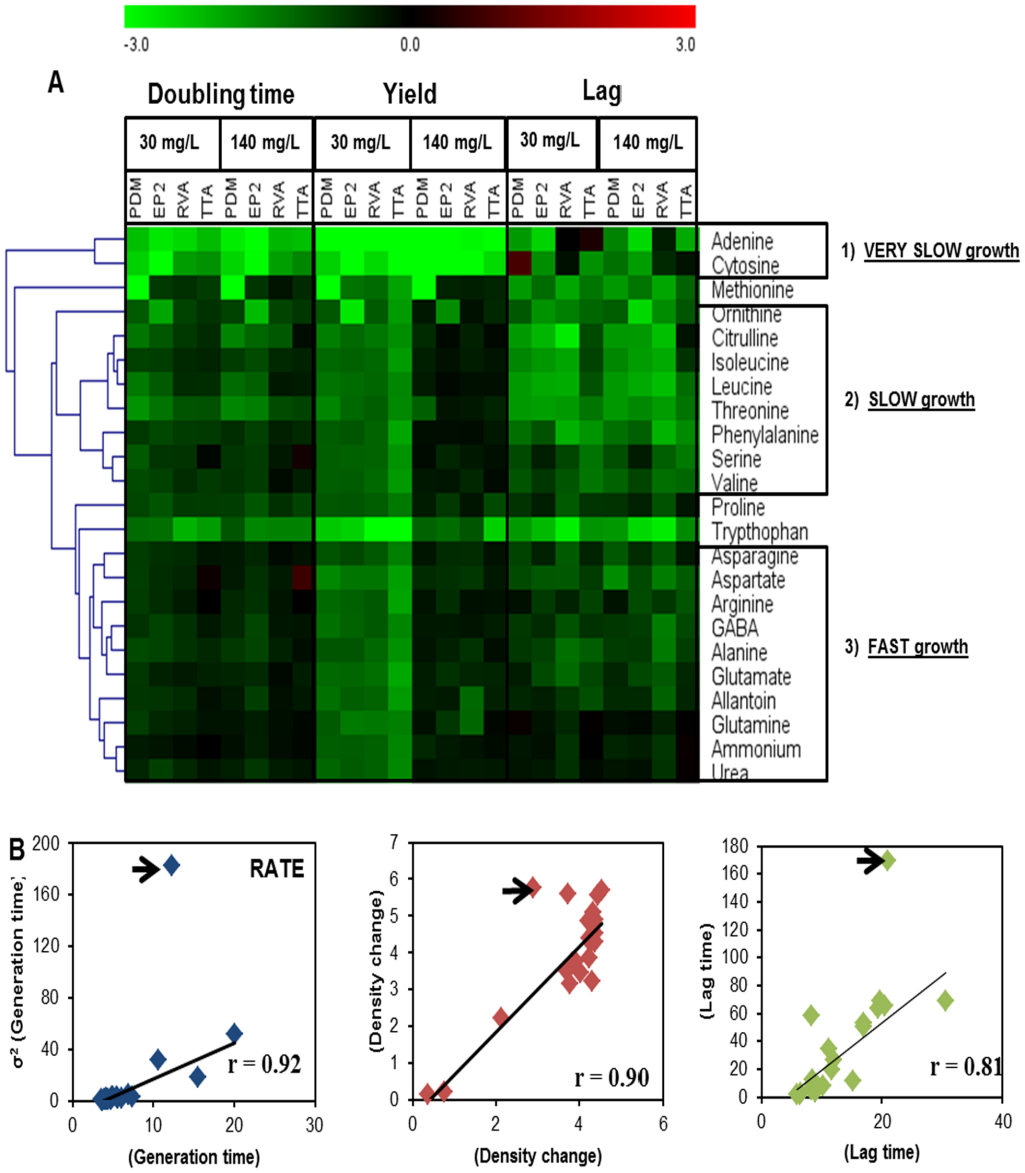
Individual Capacity of four wine strains to utilize different nitrogen sources. A) Hierarchical clustering of nitrogen sources based on the asexual fitness parameters of four wine strains. Each asexual fitness component estimate was log(2)-transformed, a mean estimate was obtained (n = 2) and this mean was normalized to the corresponding estimate (n = 2) of that strain in complete synthetic wine must (SWMc). The heatmap color reflects the normalized fitness component measure: green = inferior, red = superior and black = equal performance using a particular nitrogen source relative performance in SWMc. Based on overall performance, and considering all the fitness measures of all four strains, nitrogen sources was classified into discrete categories: “fast growth”, “slow growth” and “very slow growth”. Clustering of nitrogen sources was performed on the basis of all the fitness measures and using a Pearson correlation coefficient. Groups were clustered using group means. B) The variance in performance between the four wine strains was plotted against mean performance by considering nitrogen source and each fitness component separately. Linear regression (black line) is displayed. The squared Pearson correlation coefficient (r) is provided in the figure. Methionine (marked with an arrow), which was a clear outlier due to the inability of PDM to utilize this nitrogen source, was excluded. When including methionine, the squared Pearson correlation coefficients were: rate = 0.56, efficiency = 0.81, lag = 0.56.

**Figure 3:**
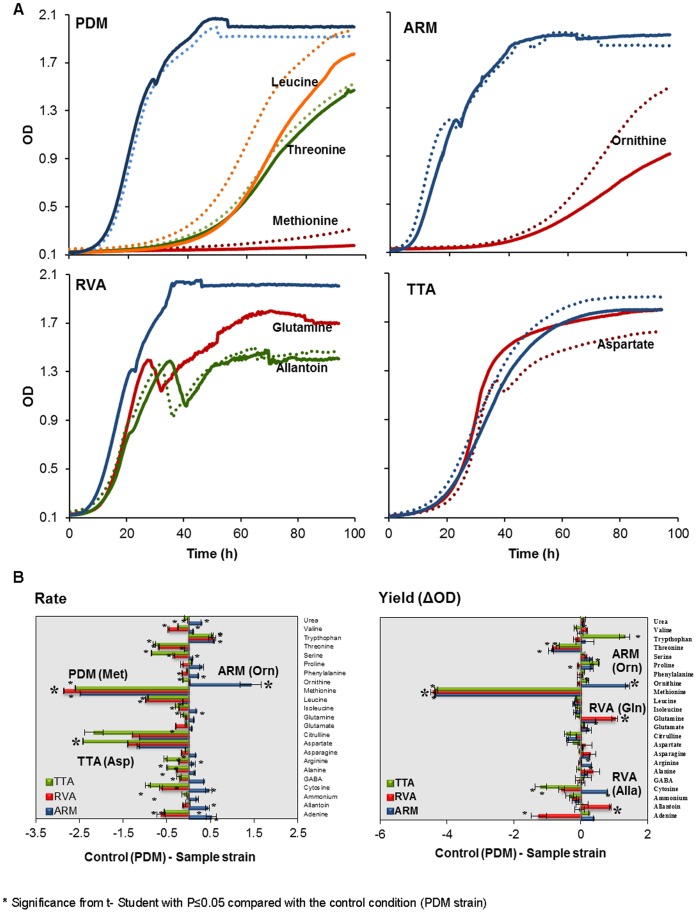
Wine strains differ in terms of their ability to use a variety of nitrogen sources. A) The growth curves of the four wine strains in those nitrogen sources were more affected in relation to the SWM control (blue line). Both replicates are displayed as full and broken lines in the same color. B) The log(2) asexual fitness component measures (generation time and yield) of TTA, RVA and ARM utilizing individual nitrogen sources were compared to the corresponding measures of PDM, log_2_(PDM)−log_2_(strain). Negative values indicate a worse performance of the PDM strain, positive values indicate better performance of the PDM strain. Error bars = SEM (n = 2).

To control for the general differences between strains, growth measures were log_2_-transformed and normalized to the corresponding fitness measure in a medium containing a complex mixture of nitrogen sources. By visualizing the relative measures of nitrogen utilization ability, we found marked differences between the wine strains in terms of their capacity to utilize different nitrogen sources (Fig. 2A). As in the ranking described in Figure 1D, grouping the nitrogen sources based on similarities in their suitability for different strains revealed three distinct clades. Clade 1 contained the consistently very poor adenine and cytosine; Clade 2 included the branched-chain and aromatic amino acids, together with arginine intermediates ornithine and citrulline and serine and threonine; Clade 3 comprised the generally good nitrogen sources asparagine, aspartate, arginine, GABA, alanine, glutamate, glutamine, ammonium, together with the animal secretion products allantoin and urea. Methionine, proline and tryptophan constituted the outliers in clustering, which imply that their profiles over all the strains and variables did not substantially resemble any other nitrogen source profile. This suggests that private mutations with little influence on other nitrogen catabolic processes underlie the variations in the utilizations of these nitrogen sources. Interestingly, the between-strain variations in nitrogen source suitability strongly and inversely correlated with mean suitability (Pearson R>0.8, p<0.001) (Fig. 2B); i.e., the most pronounced between-strains difference were observed for the worst nitrogen sources. The remarkable exception to this rule was methionine, which supported only marginal PDM growth, but emerged as an intermediate nitrogen source for other strains (Fig. 3A–B). Additionally, PDM achieved only very an inefficient utilization of threonine, and a remarkably slow utilization of leucine. Other notable strain-specific nitrogen phenotypes revealed a surprisingly poor growth of the ARM (both rate and yield) in ornithine, and diminished efficiency of RVA in glutamine and allantoin and, the fast growth of TTA using aspartate. In fact, the proliferation rate of TTA on aspartate as a sole nitrogen source exceeded the growth rate reached using the complex nitrogen mixture (Fig. 3A). Thus, aspartate was a rare exception to the widely accepted assumption of superior yeast performance in a complex mixture of nitrogen sources. Taken together, we highlight a remarkable variation in nitrogen source suitability among wine strains, with each strain harboring clear nitrogen utilization deficiencies that are potentially curable by molecular genetics.

### Wine-lab Strain Hybrids Hemizygotic for Individual Nitrogen Utilization Genes Show Mostly Unperturbed Proliferation during Nitrogen Restriction

**Figure 4:**
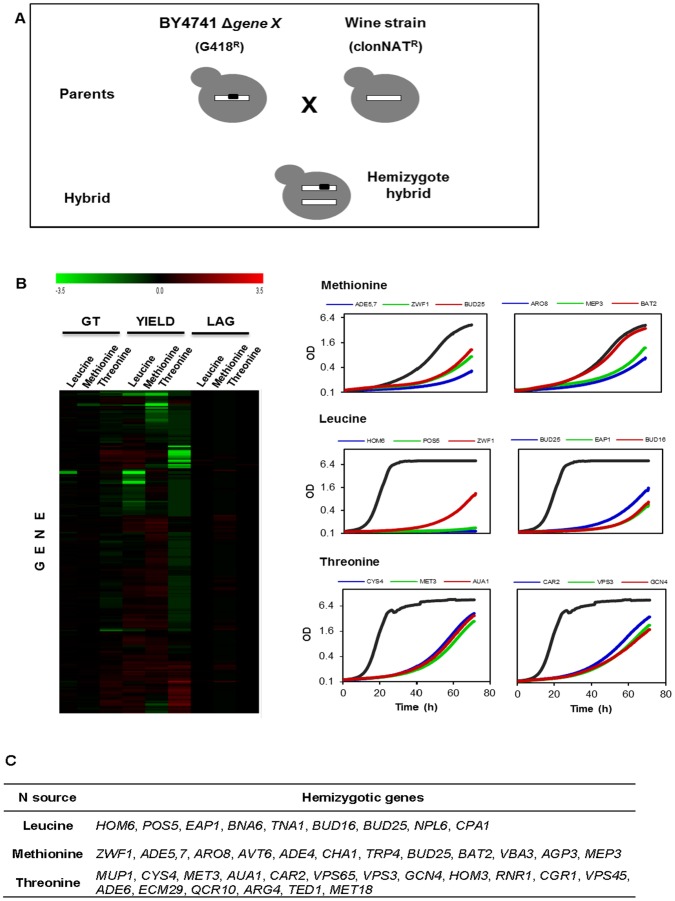
Tracing the genetic basis of wine strain variations in nitrogen utilization through designed hemizygosity in diploid hybrids. A) The hemizygosity analysis principles. The gene deletions corresponding to individual genes annotated, based on lab strain experimentation, as involved in nitrogen utilization (Table S2), were obtained from the BY4741 gene deletion collection. They were individually crossed by an automated procedure to haploid version of PDM wine strain, and diploid hemizygote hybrids were selected by reciprocal antibiotics selection. The hemizygotes, carrying only the wine strain allele of each individual targeted gene, were cultivated under nitrogen conditions of interest, and were compared to a heterozygotic diploid hybrid control carrying both the BY and wine strain allele. Deviations identify cases of haplosufficiency/haploproficiency, as well as of the wine strains alleles encoding inferior or superior nitrogen utilization. B) Capacity of PDM×BY4741 diploid hybrids that are hemizygotic for individual nitrogen utilization genes (n = 228) to utilize a variety of nitrogen sources. Log_2_ of the asexual fitness components (generation time, yield and lag phase) were normalized to the corresponding measure of the heterozygotic diploid hybrid control to produce a relative measure of nitrogen utilization capacity. Heatmap color indicates performance: green = inferior, red = superior and black = equal performance of the hemizygote to the heterozygotic control. The right panel shows the sample growth curves of the affected hemizygotic hybrids in relation to the heterozygotic diploid hybrid control (black line). Gene names indicate hemizygotic genes. C) List of wine alleles (genes) which show impaired growth in hemizygosity.

To identify the potential candidate genes harboring variations that underlie differences in nitrogen source utilization, we performed a large-scale hemizygosity analysis. To this end, 228 genes involved in nitrogen transport, catabolism, storage or regulation were selected (Table S2). The haploid single gene deletion strains in lab strain BY4741 were crossed to a haploid derivative of the PDM wine strain by a robotized procedure, and the diploid hybrids were automatically selected based on dual antibiotics resistance (Fig. 4A). The capacity of the resulting hemizygotes, which contain only the wine strain allele of each individual candidate gene, to utilize the nitrogen sources unsuitable for the wine parent was finally compared to the corresponding capacity of the heterozygote hybrid with both parental alleles intact.

The vast majority of hemizygotes closely resembled the control heterozygote (Fig. 4B). This means that retention of a single allele in the diploid hybrid was almost always sufficient to maintain an unperturbed nitrogen utilization capacity. Essentially no overlap in hemizygote defects between different nitrogen sources was observed (Fig. 4C), meaning that the effects of impairing individual nitrogen utilization functionalities had only nitrogen source-specific effects. Among the 36 hemizygotic genes showing impaired growth in methinonine, leucine and threonine (Fig. 4C), most belonged to amino acid metabolism (14 genes) and to nucleotide/nucleoside/nucleobase metabolism (5 genes). In amino acid metabolism, most alleles belonged to the sulfur amino acid metabolism (9 genes), the metabolism of the aspartate family (6) and the metabolism of glutamate (4 genes).

### Incapacity of the PDM Wine Strain to Utilize Methionine is due to Defects in*ARO8*, *ADE5,7* and *VBA3*

**Figure 5:**
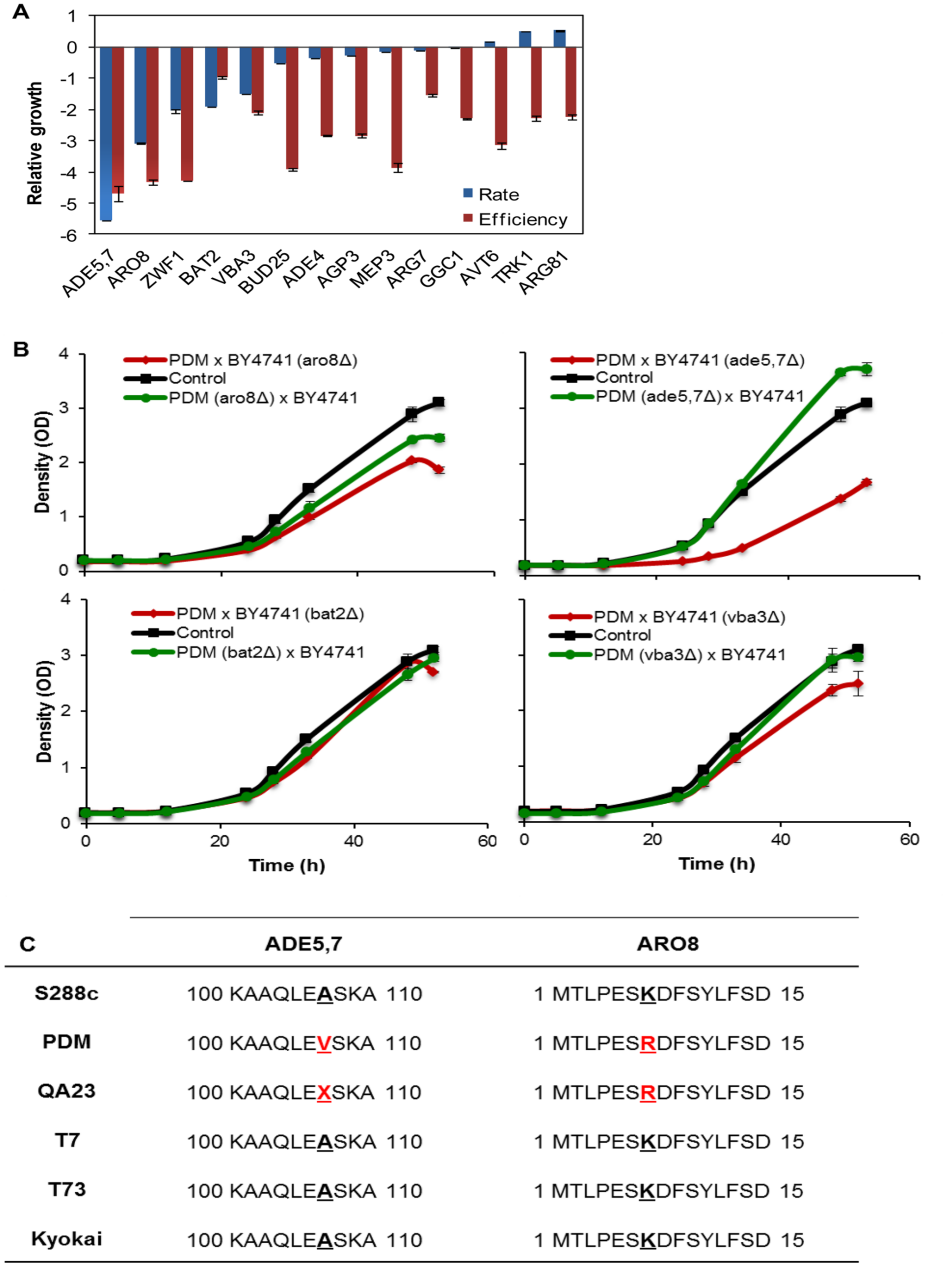
The inability of the PDM wine strain to utilize methionine is due to defects in genes*ARO8*, *ADE5,7* and *VBA3*. A) Log_2_ rate and efficiency of PDM×BY4741(ΔX) hemizygotes with deficient methionine utilization. Log_2_ measures were normalized due to the corresponding measures of the heterozygotic PDM×BY4741 hybrid control. Negative values indicate poor performance. The means of three independent replicates are displayed. B) To separate haploinsufficiency effects from defects in PDM alleles, the reciprocal hemizygotes of the four candidate genes *ARO8*, *ADE5,7*, *BAT2* and *VBA3* were constructed. PDM gene copies were removed by targeted deletions and the nitrogen utilization capacity of PDM×BY4741(ΔX) and PDM (ΔX)×BY4741 was directly compared. The heterozygote hybrid control is included as a reference. Change in optical density was measured manually. Error bars = SEM. C) Non synonymous mutations distinguishing the PDM alleles of *ADE5,7* and *ARO8* from their S288c counterparts and different wine strains. Variations were revealed by the targeted sequencing of each locus.

The genes underlying the hemizygozity associated defects in the wine-lab strain hybrid may be due to haploinsufficiency, which implies that one gene copy is not enough to maintain proliferation. Although interesting, such cases have no direct implications for wine yeast optimization for wine production. However, hemizygote defects may also be because the wine strain allele encodes an inferior gene product. Such impaired alleles are candidates for the molecular genetics-mediated optimization of wine yeast to enhance nitrogen utilization. It is possible to distinguish between haploinsufficiency and wine strain allele defects using reciprocal hemizygosity; i.e., comparing two hemizygotic diploid hybrids in which the two parental alleles of a candidate gene have been reciprocally deleted. To identify the genetic defects underlying the incapacity of the PDM wine strain to utilize methionine, we constructed reciprocal hemizygotes for four of the most promising candidate genes (Fig. 5A). The PDM methionine utilization defect was of specific interest as the aberrant utilization of methionine may contribute to the accumulation of the foul tasting and smelling sulfur intermediates in this strain [39], which may limit its value in wine production. Of the four candidate genes, *ARO8*, *ADE5,7*, *BAT2* and *VBA3*, two (*ARO8* and *BAT2)* encode the aminotransferases in the methionine salvage pathway, *ADE5,7* encodes an enzyme involved in purine biosynthesis, and *VBA3* is an amino acid transporter which facilitates the uptake of amino acids into the vacuole.

Despite comparing the methionine-based growth of the reciprocal hemizygotes in 50 mL cultures, to better mimic real wine production conditions, we were unable to confirm the defect of the *BAT2* hemizygote, identifying it as a micro-cultivation specific effect (Fig. 5B). Micro-cultivation differs from 50 mL E-flask cultures in several aspects, including lower oxygenation in the wells, potential acidification due to restricted CO_2_ efflux and restrictions on nutrient dispersion, all of which is reflected in strong activation of the general stress response system [33]. This may explain the absence of the *BAT2* phenotype in 50 mL E-flasks. In contrast, for *ARO8*, *ADE5,7* and *VBA3*, the hemizygote defects were conserved, but less pronounced than in the micro-cultivation set up. In all these cases, the hemizygotes carrying the wine strain allele performed worse than the hemizygotes carrying the lab strain allele (Fig. 5B). This established a direct causality between the PDM alleles of *ARO8*, *ADE5,7* and *VBA3* and the incapacity of PDM to utilize methionine. To identify the SNPs which potentially underlie these allelic defects, the PDM alleles were sequenced and aligned to published lab and wine strain sequences. PDM *ARO8* were found to contain six SNPs, of which only one was non synonymous, *aro8*(K7R) (Fig. 5C). *aro8*(K7R) was also present in the genetically similar wine strain QA23, but was absent in other sequenced wine strains. Of the two SNP’s in the PDM *ADE5,7* allele, A107V was non synonymous and private to PDM (Fig. 5C). The PDM *VBA3* allele was sequence identical to all the other analyzed strains, implying cis-regulatory elements underlying PDM defects. In summary, we implicated PDM *ARO8*, *ADE5,7* and *VBA3* to be causally linked to the incapacity of PDM to utilize methionine. We also identified non synonymous SNPs in *ARO8* and *ADE5,7* as good candidate targets for biotechnological efforts to alleviate the methionine defect.

## Discussion

### Nitrogen Source Utilization Differs Systematically between Wine Yeast and the Lab Strain

The fermentation rate and growth rate at all the wine yeast fermentation stages positively correlated with both the nitrogen uptake rate and the total amount of assimilated nitrogen [26], [37]. Thus, the selection, evolution or breeding of yeast strains that are able to utilize all the available nitrogen sources with a maximum rate and efficiency and a minimum lag has the potential to substantially increase the fermentation capacity in wine production. In this work, we developed a strategy to assess the ability of four commercial wine strains to utilize all the low complexity nitrogen sources supporting yeast growth and we observed a substantial quantitative variation between both strains and sources. At the most superficial level, and when only considering the growth rate, source variations approximately agreed with a crude classification into “slow”, “intermediate” and “fast” nitrogen sources, as previously established when considering lab strain Σ1278b [38]. The main determinant of this categorization is believed to be the carbon derivatives resulting from the catabolism of these compounds [38]. Whereas transamination or deamination of “fast” sources produces C-compounds directly assimilable by metabolism, the transamination of “slow” sources leads to keto-acids, which are converted into complex alcohols. However, this division into discrete categories is clearly artificial as the nitrogen sources followed an uninterrupted continuum in terms of their ability to support fast reproduction. Furthermore, a close look at the data revealed marked differences between the wine strains and the lab strain, and the most outstanding deviation was the excellent ability of urea and allantoin to support fast wine strain growth. This is somewhat surprising given that urea and allantoin are not present in grape must, but are the two main nitrogen secretion products of animals. Together with the fact that wine strains are also poorly adapted to utilize proline, the most prevalent nitrogen source in wine [3], this casts doubts about the extent to which wine yeasts are actually adapted to wine must. In general, yeast phenotypes tend to follow a population structure rather than the classifications based on source environment from which yeast is isolated [2]. This suggests that they are a consequence of either a genetic drift or a selection in ecological contexts other than the niches they currently occupy. The life history of yeast, with outcrossing being rare, and with frequent and narrow population bottlenecks, may indeed mean that it is especially prone to accumulate population-specific alleles through a genetic drift [40]. When considering the efficiency of wine strain nitrogen source utilization, the established picture of nitrogen source suitability offers even less predictive power. Many slow nitrogen sources, notably phenylalanine, leucine and citrulline, were efficiently utilized, whereas fast nitrogen sources, such as aspartate, glutamine and glutamate, were less efficiently employed. The distinction between rate and efficiency is important because, in wine production, the growth rate is typically of less importance than the final yield achieved [41]. Wine yeasts are supplied to oenologists in a dehydrated form and must be rehydrated prior to inoculation in grape must. Considering the lag time before growth takes off essentially reflects the time required to leave the latent state after rehydration and to produce sufficient metabolic and ribosomal proteins to sustain growth, which further complicates the picture. Urea, proline, ammonium and glutamine were metabolized with slight delay, whereas tryptophan, leucine, methionine and citrulline required almost one full re-adjustment day. Although the lag time in wine production has received little attention [42], it may be of substantial importance because every time delay in yeast growth is an opportunity for competing microorganisms to take over and spoil fermentation.

### Natural Variation in Nitrogen Utilization is Strain-dependent and Linked with Casual Mutations

The individual analysis of growth also revealed anomalous behaviors in different strains. The most remarkable strain-specific difference was detected in the PDM strain, which was almost completely incapable of utilizing methionine, a nitrogen source which offers otherwise better suitability to the other wine strains. Methionine is a key player of intermediary metabolism which is not only involved in protein synthesis, but is also an essential determinant of the one-carbon metabolism. Indeed in its activated form, *S*-adenosylmethionine (AdoMet) acts as the methyl donor in hundreds of transmethylation reactions of nucleic acids, proteins or lipids [43]. Thus, a defect in methionine utilization, leading to elevated intracellular pools of methionine and AdoMets, can potentially affect a large number of reactions. PDM showed several other nitrogen sources utilization defects, including inefficient utilization of threonine, and remarkably, the slow utilization of leucine. The threonine metabolism is interconnected with the methionine metabolism by the common intermediate O-acetyl L-homoserine, and several enzymes of the biosynthesis pathways are regulated by methionine or its derivatives [44]. Thus, it is not unreasonable to speculate that the methionine and threonine defects may be genetically and molecularly linked. In fact, most hemizygote strains which showed a growth defect in either of these nitrogen sources harbored wine single alleles of the sulfur amino acid metabolism (Fig. 4C). The PDM strain, and its commercial derivatives, is one of the most important genotypes in the wine industry despite its high H_2_S production in certain wine fermentation circumstances. H_2_S is a necessary intermediary in the synthesis of sulfur amino acids from sulfate. However, if not catabolized, it probably becomes a major wine production problem because of its poor organoleptical properties. Cordente *et al*. [39] obtained low H_2_S-producing strains deriving from the commercial PDM by random mutagenesis. These low H_2_S-producing strains harbored specific mutations in the *MET10* and *MET5*, which encoded the catalytic α- and β-subunits of the sulfite reductase enzyme, and they were auxotroph for methionine. Besides the deficiencies of the PDM strain, we also found nitrogen source utilization defects in all three commercial strains considered. Thus, there are ample opportunities for optimizing the nitrogen source utilization capacity of all these strains to potentially improve their suitability for industrial wine fermentation. Defects were private to each strain, meaning that they are unlikely to be the products of adaptations to the industrial process *per se*. This is important because their correction should not elicit any immediate negative influences on other phenotypes of industrial importance through the antagonistic pleiotropy relating to the gene products involved. It also suggests a possible way forward to construct commercial strains that lack these deficiencies to help face the challenges of GMO restrictions that preclude targeted genetic manipulations. As defects are caused by recessive loss-of-function mutations, which appear to be the source of the vast majority of phenotypic variations in yeast [40], the hybridization of haploid derivatives of different commercial strains to yield fully heterozygotic diploids should compensate for the respective genetic defects through reciprocal masking. Strains readily sporulated, and the mixing of spores from two different backgrounds should result in a fraction of hybrid diploids that can be selected for their phenotypic superiority in terms of nitrogen traits.

### Defects in the PDM Alleles of*ARO8*, *ADE5,7* and *VBA3* Underlie the Inability to Utilize Methionine

However, any effort into strain optimization for wine production would benefit from prior knowledge of the underlying genetics. The wine strains herein investigated do not easily lend themselves to QTL mapping; instead, we utilized a naive, large-scale hemizygote approach to test whether any of 228 genes known to be involved in nitrogen utilization can harbor wine strain polymorphisms causing phenotypic deficiencies. Although this approach allows for the rapid generation of large sets of hemizygotes, through the mating of the BY deletion collection to a haploid wine strain derivative, it does not immediately distinguish between defects to haploinsufficiency, i.e., retention of only a single gene copy, and defects due to polymorphisms. However, given the scarcity of hemizygote defects, <5% (10 of 228) in the case of the PDM methionine defects, despite the gene products being directly involved in the molecular process targeted, this should be of less concern. We conclude that even in challenging nitrogen-limited environments, retention of a single copy of nitrogen metabolism-related genes is almost always enough to maintain nitrogen-dependent functionalities unperturbed. This has several important implications. First, it supports and extends the observations from lab strains in optimal environments [45] that haploinsufficiency is remarkably rare. This also agrees with functional alleles tending to completely dominate non functional alleles in yeast hybrids [40]. This is not because of the compensatory induction of the remaining gene copy, but because half the normal production of a gene product suffices to support proliferation [46]. It also suggests that such a naive approach to understanding genetic variation, circumventing QTL mapping and fine mapping of QTLs, and focusing directly on allele phenotype interactions, are a viable alternative in yeast genetics. This shifts the burden of work from strain construction to phenotypic screening.

We selected 4 of the 14 gene candidates for causing the methionine defect, and found that 3 of these corresponded to true defects in wine strain alleles. Of the three alleles, *ARO8*, *ADE5,7* and *VBA3*, herein identified as contributing to the methionine utilization deficiency of PDM, *ARO8* is the only one with a clear, direct connection to methionine metabolism. This gene encodes a transaminase of the methionine salvage pathway, together with Bat2, herein initially identified, but not confirmed as a candidate, and Aro9 and Bat1. At low methionine concentrations, these enzymes transfer the amino group from an amino acid to 2-oxo-4- methylthiobutanoate, resulting in the production of a ketoacid and methionine [47]. The methionine salvage pathway comprises a set of complex reactions that allows the direct synthesis of methionine from 5′-methylthioadenosine (MTA) [48]. Moreover, the first step of this pathway also releases adenine in the metabolism of which the other defective allele, *ADE5,7,* is also involved. Although the salvage pathway has primarily been studied in the methionine synthesis context, it is likely that the use of methionine as a sole nitrogen source, and therefore as a sole amino donor, reverses the flow of this reaction. *ARO8* would then be a key amino transferase by shifting nitrogen from methionine to keto acids in the first step of the methionine catabolic pathway. In this case, the other three transaminases catalyzing this reaction (Aro9, Bat1 and Bat2) are apparently unable to compensate for the *ARO8* defect, potentially because of the various affinities for different ketoacids. It is quite plausible that the *ARO8* defect correlates to excess H_2_S production. Dysfunction in the conversion of methionine into other amino acids should increase intracellular methionine. An elevated pool of internal methionine leads to increased homocysteine, and parts of this excess can be converted into O-acetyl-serine via Met17, with H_2_S emerging as a secondary product of this reaction.

The connection of *VBA3* and *ADE5,7* to methionine utilization defects are less clear. *VBA3* is a vacuolar transporter of basic amino acids lysine, histidine and arginine [49], but likely not of methionine, although it has not been tested. If facilitates the vacuolar storing of these amino acids at high concentrations; e.g. >20x the cytoplasmic concentration in terms of arginine [50], when they are in excess, but it is not known whether it can catalyze the mobilization of these storages when deficiencies emerge. It can be speculated that a deficiency in such vacuolar mobilization of these aminoacids, when their cytoplasmic production from methionine is impaired, can be the mechanistic cause of the wine strain Vba3 allele’s contribution to poor methionine growth. Ade5,7 is a bifunctional enzyme that facilitates nucleotide biosynthesis when sufficient nucleotides are not supplied externally. The methionine salvage pathway is also a supplier of purines. Thus, an irregular function of this pathway can increase the synthesis requirement through the *de novo* biosynthesis of purine nucleotides in which the mutated *ADE5,7* allele operates. Interestingly, Ade5,7 deletion strains are also highly sensitive to the sulfite-like metal ion tellurite, a phenotype that is otherwise strongly associated with defects in the methionine metabolism [51]. This supports the existence of links to this metabolic pathway.

## Supporting Information

Table S1 **Primers used in this study.**
(DOC)

Table S2 **Distribution in GO-functional categories.**
(DOC)
