# Supplementary material for: Genetic Basis of Variations in Nitrogen Source Utilization in Four Wine Commercial Yeast Strains
Source: PLoS One. 2013 Jun 24;8(6):e67166. doi: 10.1371/journal.pone.0067166 (PMC3691127; doi:10.1371/journal.pone.0067166)
Supplement: Table S1 — Primers used in this study. (DOC) [file pone.0067166.s001.doc]

**Table S1**. Primers used in this study

| **Gene** | **Name** | **Oligonucleotide sequence (5’-3’ end)** |
| --- | --- | --- |
| *HO* disruption cassette * | HO-S1 | AGACATCGCAAACGTCACGGCTAACTCTTACGTTATGTGCGCAGATGGCTCGTACGCTGCAGGTCGACA |
|  | HO-C2 | ACTCTTATGAGGCCCGCGGACAGCATGAAACTGTAAGATTCCGCCACATTACTAGTGGATCTGATATC |
| *HO* PCR verification | HOc-F | GAGGTTTGCAGAAGCTTGTTGA |
|  | HOc-R | TTGGCGTATTTCTACTCCAGCAT |
| Mating type verification | MAT | AGTCACATCAAGATCGGTTATGG |
|  | MATF | GCACGGAATATGGGACTACTTCG |
|  | MATa | ACTCCACTTCAAGTAAGAGTTTG |
| *ARO8* disruption cassette * | ARO8-Ft | AACCCTGCAGTTGATACAGACATTGAATAGGACAACCGATCGTTACTATCCGTACGCTGCAGGTCGACG |
|  | ARO8-Rt | CGTACGTCCTTTTTTCACCTTATATATATTCTTCCAACGTATTTACCTCTACTAGTGGATCTGATAT |
| *BAT2* disruption cassette * | BAT2-Ft | AAAATTTTAGAAATTTAAGGGAAAGCATCTCCACGAGTTTTAAGAACGATCGTACGCTGCAGGTCGACG |
|  | BAT2-Rt | AGTTTTATTCTTTTTAACTTTTAATTACTTTACGTAGCAATAGCGATACTACTAGTGGATCTGATAT |
| *ADE5,7* disruption cassette * | ADE5,7-Ft | TATTACTTTCTTAATCATAGCTTAAGAGAACCATTCTCCCTCCCCTCACACGTACGCTGCAGGTCGACG |
|  | ADE5,7-Rt | TAATATATGTACGCGCATATATGAATCTATTATAAAGTTAATATTGTTGAACTAGTGGATCTGATAT |
| *VBA3* disruption cassette * | VBA3-Ft | CAATACTTATTTTTGAAGCCGGATCCCTAATTGCTGCCCTTGCCTCTTCACGTACGCTGCAGGTCGACG |
|  | VBA3-Rt | GTTGAAGTCTGTATAAAAGGCGAAAAAAATAAAATGAAAATAAGAAAATAACTAGTGGATCTGATAT |
| *ARO8* PCR verification | ARO8-Fc | TGGCTCATATACACCATCCA |
|  | ARO8-Rc | GGAGATTCATGGTACCAGACA |
| *BAT2* PCR verification | BAT2-Fc | TCACGACCTAGCATACCACTA |
|  | BAT2-Rc | GATAGGCCAGCACTAGATGA |
| *ADE5,7* PCR verification | ADE5,7-Fc | CCGTTGTTCATACCGTGACTA |
|  | ADE5,7-Rc | GCCAGTAAAACCTGCTGAAA |
| *VBA3* PCR verification | VBA3-Fc | TACCTAGAACTGATCAGACCA |
|  | VBA3-Rc | CTTATTGTGAAAGGTGTTTGA |
| *ARO8* sequence | ARO8-Fs1 | CGTTACTATCATGACTTTACC |
|  | ARO8-Rs1 | TACCTCTCTATTTGGAAATAC |
|  | ARO8-Fs2 | CTGGTGCTCCTAAACCAAAGT |
|  | ARO8-Rs2 | TCATGCAAACTAAGTAAGGC |
| *BAT2* sequence | BAT2-Fs1 | GCATCTCCACGAGTTTTAAG |
|  | BAT2-Rs1 | CGTAGCAATAGCGATACTTCA |
|  | BAT2-Fs2 | TATATGTCATTTGCTGCCCTG |
|  | BAT2-Rs2 | TTCATGGTGCCGACTTCAGTA |
| *ADE5,7* sequence | ADE5,7-Fs1 | TAAGAGAACCATTCTCCCTC |
|  | ADE5,7-Rs1 | GATTAGTAAAGCTTAGTTCCG |
|  | ADE5,7-Fs2 | GGCAAGCAGGTTGTGATAGAA |
|  | ADE5,7-Rs2 | GAATTTTGGAAATGGACGCA |
|  | ADE5,7-Fs3 | GCCACCGATTCTTTATTGACC |
|  | ADE5,7-Rs3 | TTAATACAGCACCAACAGCGG |
| *VBA3* sequence | VBA3-Fs1 | GCCTCTTCAATGAATATGCTC |
|  | VBA3-Rs1 | TAACTGCGCTACTTGTCTTCT |
|  | VBA3-Fs2 | CGATAAATTCAACCCGGA |
|  | VBA3-Rs2 | GTGGTTTAACTAAACCGAGC |

* Underlining indicates homology to the *nat1* cassette from plasmid pAG25. The remaining sequences of the primers are homologous to the flanking region of the *HO* open-reading frame

PCR, polymerase chain reaction
