# Supplementary material for: Genetic Basis of Variations in Nitrogen Source Utilization in Four Wine Commercial Yeast Strains
Source: PLoS One. 2013 Jun 24;8(6):e67166. doi: 10.1371/journal.pone.0067166 (PMC3691127; doi:10.1371/journal.pone.0067166)
Supplement: Table S2 — Distribution in GO-functional categories. (DOC) [file pone.0067166.s002.doc]

**Table S2**. Distribution in GO-functional categories

| **Functional categories** | **Genes** |
| --- | --- |
| **METABOLISM** |  |
| **Amino acid metabolism** |  |
| Glutamine | *CPA2, ZWF1, CPA1* |
| Glutamate | *GDH3, GLT1, GDH2, PUT2, AAT1, MEU1, AAT2, PUT1, ACO1, GAD1, GDH1* |
| Arginine | *VBA3, ARG82, ARG5,6, ARG4, ARG2, ARG3, CPA2, PUT1, CAR2, ARG81, ARG80, VBA1, ARG1, ORT1, CPA1, CAR1, ARG7* |
| Aspartate | *ASP1, ARG4, AAT1, AAT2, ARG1* |
| Threonine | *CHA1, THR4, HOM2, HOM3, ILV1, THR1, HOM6* |
| Methionine | *HOM2, SAM2, HOM3, MET6, MET10, CYS4, THR1, MET18, MET3, CBF1, ECM17, HOM6, MET14, MER1, SAM1, MET17,* |
|  | *ADI1, MET2, YPR118w, MET16, MET5* |
| Lysine | *LYS2, VBA3, LYS5, HOM6, VBA1* |
| Serine | *CHA1, HOM2, HOM3, SER3, ILV1, SER2, THR1, SER33, HOM6, MET2, SER1* |
| Phenylalanine | *TYR1, ARO1, ARO10, ARO2, ARO8, ARO9, PHA2, ARO7* |
| Tryptophan | *TYR1, TRP1, ARO1, TRP4, TRP2, TRP5, ARO2, ARO8, ARO9, BNA2, TRP3, BNA5, ARO7* |
| Pyruvate family | *ILV6, ARO10, ILV1, THR1, BAT2* |
| Regulation of amino acid metabolism | *ARG82, GCN4, AUA1, ARO8, ARO9, MET28, DAL81, CBF1, ARG81, ARG80* |
| **Nitrogen metabolism** | *DUR1,2, DAL1, DAL2, DAL7, DAL3, DAL82, CPS1, ALT1* |
| Regulation of nitrogen metabolism | *MET8, UGA3, NPR2, GLN3, AUA1, GAT1, VID30, UGA1, MET28, DAL81, DCG1, GZF3, CBF1, DAL80, ARG81, ARG80,* |
|  | *MKS1, NPR1, URE2, CAR1, NPR3* |
| **Purin nucleotide metabolism** | *ADE1, DUR1,2, HIS4, HPT1, ADE8, ADE5,7, ADE6, ADE3, DAL1, DAL2, DAL7, DAL3, MEU1, ADE16, APT1, AMD1,* |
|  | *ADE17, ADE4, ADE12, ADE2, SER1, ADY2, RNR1, DAL81, MET3, MKS1, DAL82, FCY1* |
| **Phosphate metabolism** | *HIS4, KCS1, PPH3, ARO1, ARG82, HOM3, ARG5,6, FAB1, SER2, THR1, TOR1, MET14, VPS34, CTK3, NPR1, POS5* |
| **Carbohydrate metabolism** | *ADH5, ARO4, ILV6, ADY2, SFA1, ARO3, ARO1, ARO10, ADE8, SAM2, YEA4, GSY1, ADH4, UGA1, PDC6, ADE3, GND1,* |
|  | *DAL7, GON7, ADE16, PDC1, PDC5, SAM1, ACD1, ADH3, ADE17, ADH2, MKS1, ZWF1, ATO2, TKL1, YPR118W* |
| **Lipid metabolism** | *PDX3, KCS1, ARG82, DPL1, FAB1, TOR1, OPI3, VPS34, ANT1* |
| **Vitamins metabolism** | *PDX3, THR4, THI3, BNA6, ARO2, LYS5, ADE3, BNA1, BNA2, MET1, BNA5, RIB4, SER1, MET7, POS5* |
| **TRANSPORT** | *AVT5, TAT1, VMA2, AGP1, GGC1, UGA4, PEP7, ATO3, VPS3, GNP1, CAN1, FCY22, AVT6, AGP3, LOC1, VPS45, MUP1,* |
|  | *MEP1, BTN2, TNA1, DUR3, MUP3, DAL4, TRK1, AVT1, DAL5, AVT3, GAP1, VPS9, NPL6, AVT4, MEP2, ALP1, TAT2,* |
|  | *PUT4, FRE3, FIT2, VPS28, DIP5, AGC1, VMA13, MEP3, OPT2* |
| **ENERGY** | *QCR10* |
| **TRANSCRIPTION** | *STP2, ISY1, TIF2, ROX3, TRM1, CDC40, CGR1, PRP18, STB5* |
| **CELL RSUE AND DEFENSE** | *SOD1, ROX3, HMF1* |
| **PROTEIN FATE** | *NTA1, VAM6, VMA1, HPA3, ECM29* |
| **PROTEIN SYNTHESIS** | *EAP1, CAF20, TIF2, YOR302W* |
| **CELL CYCLE AND PROCESSING** | *MSC1, BUR2, CDC40, HPA3* |
| **BIOGENESIS OF CELLULAR COMPOUNDS** | *ECM25, VAM6, HPA3, CGR1, ECM29* |
| **CELL TYPE DIFFERENTIATION** | *BUD16* |
| **Unclassified proteins** | *YER091C-A, YER137C, APT2* |
|  | *IBA57, MRI1, VPS65, YDR008C, YDR442W, BUD25, YER068C-A,TED1* |
